# Supplementary material for: The Systems Biology Research Tool: evolvable open-source software
Source: BMC Syst Biol. 2008 Jun 29;2:55. doi: 10.1186/1752-0509-2-55 (PMC2446383; doi:10.1186/1752-0509-2-55)
Supplement: Additional file 1 — SBRT Archive. An archive of the current version of the Systems Biology Research Tool. [file 1752-0509-2-55-S1.zip › sbrt-1.4.0/doc/developers_guide/api/sbrt/kernel/math/Polynomial.html]

Polynomial


|  |  |  |
| --- | --- | --- |
| **Overview** \| **Package** \| **Class** \| **Use** \| **Tree** \| **Deprecated** \| **Index** \| **Help** | |  |
| **PREV CLASS**   **NEXT CLASS** | **FRAMES**    **NO FRAMES**     **All Classes** |  |
| SUMMARY: NESTED \| FIELD \| CONSTR \| METHOD | DETAIL: FIELD \| CONSTR \| METHOD |  |


---


## sbrt.kernel.math Interface Polynomial<V>

**Type Parameters:**: `V` - the variable type.

**All Superinterfaces:**: MathExpr<V>

**All Known Subinterfaces:**: LinearComb<V>, QuadraticExpr<V>

**All Known Implementing Classes:**: AbstractLinearComb, AbstractPolynomial, HashLinearComb

---

``` public interface Polynomial<V> extends MathExpr<V> ```

This interface is used to represent polynomials.

A polynomial is a type of mathematical expression. It is the
sum of a numerical constant and a set of terms. Each term
contains a numerical
coefficient and at least one variable. Each variable in a term
can be raised to a non-negative whole number power. These basic
facts about polynomials have been captured in this interface.

**Author:**
:   This interface was written and documented by
    Jeremiah Wright while in the Wagner lab.

---

| **Nested Class Summary** | |
| --- | --- |
| `static interface` | `Polynomial.Term<V>`             This interface is used to represent the terms of a polynomial. |


| **Method Summary** | |
| --- | --- |
| `boolean` | `equals(java.lang.Object obj)`             Indicates if the provided object is equal to this polynomial. |
| `double` | `getConstant()`             Returns the numerical constant of this polynomial. |
| `Polynomial.Term<V>[]` | `getTermArray()`             Returns an array containing all of the terms of this polynomial. |
| `int` | `hashCode()`             Returns a content-based hash code for this polynomial. |
| `Polynomial<V>` | `plugInNumbers(java.util.Map<?,java.lang.Double> variableValues)`             Plugs in the provided numerical values to produce a new polynomial. |
| `int` | `terms()`             Returns the number of terms contained in this polynomial. |
| `java.lang.String` | `toString()`             Returns an appropriate string representation of this polynomial. |

**Methods inherited from interface sbrt.kernel.math.MathExpr**

`getVariables, hasAllVariables, hasVariable, hasVariables, variables`

**Method Detail**

### plugInNumbers

```
Polynomial<V> plugInNumbers(java.util.Map<?,java.lang.Double> variableValues)
```

:   Plugs in the provided numerical values to produce a new
    polynomial.

    If some of the keys in the provided map are not variables
    in this expression, their provided value is simply ignored.
    If a value is provided for each variable in this polynomial,
    the resulting polynomial will not contain any
    terms of its own, only a constant.

    :   **Specified by:**: `plugInNumbers` in interface `MathExpr<V>`
    :   **Parameters:**: `variableValues` - objects mapped to numerical values. **Returns:**: the polynomial obtained by "plugging in" the provided numerical values in the standard mathematical sense.

---


### getConstant

```
double getConstant()
```

:   Returns the numerical constant of this polynomial.

    :   **Specified by:**: `getConstant` in interface `MathExpr<V>`
    :   **Returns:**: the numerical constant of this polynomial.

---


### getTermArray

```
Polynomial.Term<V>[] getTermArray()
```

:   Returns an array containing all of the terms of this
    polynomial.

    :   **Returns:**: an array containing all of the terms of this polynomial.

---


### terms

```
int terms()
```

:   Returns the number of terms contained in this polynomial.
    The numerical constant is not considered a term.

    :   **Returns:**: the number of terms contained in this polynomial.

---


### hashCode

```
int hashCode()
```

:   Returns a content-based hash code for this polynomial.

    :   **Specified by:**: `hashCode` in interface `MathExpr<V>` **Overrides:**: `hashCode` in class `java.lang.Object`
    :   **Returns:**: a content-based hash code for this polynomial

---


### equals

```
boolean equals(java.lang.Object obj)
```

:   Indicates if the provided object is equal to this
    polynomial.

    For two polynomials to be equal, they must contain
    the same numerical constant and terms.

    :   **Specified by:**: `equals` in interface `MathExpr<V>` **Overrides:**: `equals` in class `java.lang.Object`
    :   **Parameters:**: `obj` - the object to be tested for equality with this mathematical expression. **Returns:**: `true` if this polynomial is equal to the provided object; `false` otherwise. **See Also:**: `Polynomial.Term.equals(Object)`

---


### toString

```
java.lang.String toString()
```

:   Returns an appropriate string representation of this
    polynomial.

    :   **Specified by:**: `toString` in interface `MathExpr<V>` **Overrides:**: `toString` in class `java.lang.Object`
    :   **Returns:**: an appropriate string representation of this polynomial.


---


|  |  |  |
| --- | --- | --- |
| **Overview** \| **Package** \| **Class** \| **Use** \| **Tree** \| **Deprecated** \| **Index** \| **Help** | |  |
| **PREV CLASS**   **NEXT CLASS** | **FRAMES**    **NO FRAMES**     **All Classes** |  |
| SUMMARY: NESTED \| FIELD \| CONSTR \| METHOD | DETAIL: FIELD \| CONSTR \| METHOD |  |


---
